# Intervention vignettes as a qualitative tool to refine complex intervention design

**DOI:** 10.1186/1745-6215-14-S1-O55

**Published:** 2013-11-29

**Authors:** Pat Hoddinott, Heather Morgan, Gill Thomson, Nicola Crossland, Leone Craig, Jane Britten, Shelley Farrar, Rumana Newlands, Kirsty Kiezebrink, Joanne Coyle

**Affiliations:** 1 University of Stirling, Stirling, UK; 2 University of Aberdeen, Aberdeen, UK; 3 University of Central Lancashire, Preston, UK

## Background

In trial design, decisions are made about which intervention components/processes to standardise and which remain flexible to maximise utility and/or effectiveness. The intervention-context-system fit for complex interventions impacts on trial recruitment, delivery and outcomes. Survey vignettes and discrete choice experiments are quantitative researcher led approaches which focus on a few measurable attributes. Our aim was to explore the utility of qualitative vignettes as a methodological tool allowing service users/providers to contribute to intervention design.

## Methods

A case series of four acceptability and feasibility studies (qualitative interviews and focus groups) with service users and providers. Data were collected at different pre-trial stages: i) vignettes of studies in a systematic review of incentives for breastfeeding and smoking cessation in pregnancy, subsequently modified following emergent qualitative analysis; ii) emergent vignettes in the last of up to 8 serial qualitative interviews investigating infant feeding behaviour, following a systematic review showing poor generalizability of effective interventions in the UK context; iii) intervention vignettes of an effective intervention (groups for weight management) to refine the design for a new population (women treated for breast cancer) and iv) emergent intervention vignettes explored at a second interview with obese older adults.

## Findings

Illustrations of how qualitative vignettes can complement quantitative design tools will be presented.

## Conclusion

Carefully constructed qualitative vignettes combining known effective and emergent promising intervention aspects can optimise trial design. When talking service users and providers through a potential intervention, different perspectives emerge compared with responses to closed or more abstract questions.

